# A Decision Tree Classification Algorithm Based on Two-Term RS-Entropy

**DOI:** 10.3390/e27101069

**Published:** 2025-10-14

**Authors:** Ruoyue Mao, Xiaoyang Shi, Zhiyan Shi

**Affiliations:** School of Mathematical Sciences, Jiangsu University, Zhenjiang 212013, China; 13952850523@163.com (R.M.); xshi0959@gmail.com (X.S.)

**Keywords:** classification, decision tree, generalized entropy, split criteria

## Abstract

Classification is an important task in the field of machine learning. Decision tree algorithms are a popular choice for handling classification tasks due to their high accuracy, simple algorithmic process, and good interpretability. Traditional decision tree algorithms, such as ID3, C4.5, and CART, differ primarily in their criteria for splitting trees. Shannon entropy, Gini index, and mean squared error are all examples of measures that can be used as splitting criteria. However, their performance varies on different datasets, making it difficult to determine the optimal splitting criterion. As a result, the algorithms lack flexibility. In this paper, we introduce the concept of generalized entropy from information theory, which unifies many splitting criteria under one free parameter, as the split criterion for decision trees. We propose a new decision tree algorithm called RSE (RS-Entropy decision tree). Additionally, we improve upon a two-term information measure method by incorporating penalty terms and coefficients into the split criterion, leading to a new decision tree algorithm called RSEIM (RS-Entropy Information Method). In theory, the improved algorithms RSE and RSEIM are more flexible due to the presence of multiple free parameters. In experiments conducted on several datasets, using genetic algorithms to optimize the parameters, our proposed RSE and RSEIM methods significantly outperform traditional decision tree methods in terms of classification accuracy without increasing the complexity of the resulting trees.

## 1. Introduction

Decision tree algorithms are classic machine learning methods used for handling classification and regression problems. Despite being around for nearly half a century, they remain active in various fields of machine learning, thanks to their strong performance characteristics, such as high accuracy, simple algorithmic processes, and excellent model interpretability [1].

The principle of decision tree algorithms can be summarized as a recursive process that repeatedly selects features to partition the dataset, with the goal of reducing the disorder or complexity of the variable to be classified or regressed as much as possible. For new data points, the algorithm returns the value of the variable to be classified or regressed based on the selected features, thus completing the classification or regression task [2]. Therefore, the questions of how to measure the disorder or uncertainty of variables, how to find features to divide the dataset, and what conditions should trigger the termination of recursion become key issues when building a tree. To address these challenges in tree construction, many well-known decision tree algorithms have been developed. For example, in the ID3 algorithm proposed by Quinlan, the concept of Shannon entropy from information theory is used to describe the disorder of classification variables. Subsequently, he also introduced an improved version of the C4.5 algorithm, which not only incorporated the Gini index as a new measure of classification variable uncertainty but also introduced the concept of information gain ratio [2,3]. Breiman’s CART decision tree algorithm combines both classification and regression problems and introduces stopping criteria for dividing trees, allowing for control over the complexity of the trees and enabling them to handle regression problems [3]. As decision tree algorithms continue to mature, they are increasingly applied to disciplines like management science, medicine, biology, etc. In combination with other statistical methods, decision trees can also achieve more outstanding performance [4,5,6,7]. Kumar et al. proposed a hybrid classification model combining support vector machines with decision trees that significantly improves computational efficiency without sacrificing accuracy [8]. Bibal et al. designed a method called DT-SNE for visualizing high-dimensional discrete data, which combines the visualization capabilities of t-SNE with the interpretability of decision trees [9].

However, decision tree algorithms themselves also have some drawbacks. Rokach et al. point out that decision trees often perform poorly when faced with attributes involving complex interactions, and duplication problems are an example of this deficiency [10]. When faced with imbalanced datasets, decision trees may also produce fragmentation problems, which result in low predictive credibility due to too few samples before and after splitting, or even overfitting [11]. The Random Forest algorithm, introduced by Breiman, is an ensemble algorithm based on decision trees. Compared to decision trees, it has stronger identification capabilities for complex attributes and better accuracy and robustness to noise [12]. However, decision trees as base learners still have some drawbacks. When constructing single decision trees, the algorithm only considers the best decision at each step, exhibiting myopia due to its greedy nature. Both decision trees and random forests typically construct trees based on specified criteria for category complexity reduction, such as entropy or Gini Index, limiting decision flexibility. Moreover, numerous practical applications show that different criteria lead to varying levels of performance among decision tree algorithms, indicating that there is no one-size-fits-all segmentation standard for building trees suitable for all datasets and performing well.

To achieve higher flexibility in decision trees, we aim to introduce some generalized entropy measures for complexity that allow the segmentation criteria based on these entropies to adjust according to different datasets. Many generalized entropies have been proposed, such as Rényi entropy, Tsallis entropy, and *r*-type entropy [13]. These are single-parameter generalized entropies, with Shannon entropy being a special case for specific parameter values. Similarly to Shannon entropy, the Gini index is a value calculated based on a probability distribution, used to measure sample category impurity [14]. Wang et al. pointed out that Tsallis entropy possesses better properties [15]. Under different parameter values, Tsallis entropy can converge to both Shannon entropy and the Gini index. Therefore, Tsallis entropy not only unifies Shannon entropy and the Gini index within a single framework but also allows for searching other potentially more suitable parameter values for the current dataset. Experiments have shown that this improvement significantly enhances the accuracy of decision trees when handling classification tasks. Optimal construction of binary decision trees has been proven an NP-hard problem, meaning that it is difficult to build an optimal decision tree in polynomial time [16]. Therefore, the construction process of most decision trees is greedy, where at each step, the seemingly optimal option is chosen to generate the tree structure. Wang et al. proposed the two-term information measures based on Tsallis entropy and design a less-greedy tree construction algorithm TEIM. Experiments on various datasets demonstrate that this algorithm outperforms traditional decision trees constructed using a single information measure in terms of accuracy and robustness while partially overcoming the short-sightedness issue of decision trees. It is also worth noting that this approach of optimizing decision trees by modifying the splitting criterion does not alter the tree generation structure. Consequently, neither the time complexity nor the search complexity of tree construction undergoes any change [17].

This paper presents an improvement to this less-greedy tree construction method. Inspired by generalized entropy and two-term information methods, we seek a more general unified framework through considering a broader range of entropy functions. At the same time, we re-examine the two-term information method, treating it as a split criterion with penalties to further improve decision tree algorithm performance. In summary, our innovations are as follows:

- We introduce a two-parameter generalized entropy framework—the rs‐entropy, which unifies Rényi entropy, Tsallis entropy, and the *r*-type entropy under a common framework—to enhance decision tree generalization ability further [18].
- Utilizing the second term in the two-term information method as a penalty term in the split criteria and introducing a penalty coefficient to the said term, leading to increased interpretability and adaptability to diverse datasets.
- Incorporation of Genetic Algorithm (GA) for rapid determination of parameter values within our improved decision tree construction algorithm. This optimization approach ensures effective parameter tuning for fast and accurate tree generation.
- In terms of evaluating model performance, we conducted time and space complexity analyses in comparison with traditional algorithms. Meanwhile, we combined the performance of each model on the test set with non-parametric tests to assess the significance of differences in model superiority.

The remainder of this paper will focus on designing our improved decision tree algorithm. In Section 2, we discuss relevant concepts of information entropy serving as the theoretical foundation for our algorithm. Section 3 delves into details regarding improvements made to existing algorithms along with the corresponding steps involved. Lastly, in Section 4, we evaluate our enhanced model through comparison with conventional methods, highlighting its superiority.

## 2. Information Entropy Theory

In this chapter, we will introduce a theoretical framework for information entropy, which is a measure of uncertainty or complexity in the case of a random variable. This part will be used as the theoretical support of the decision tree algorithm later.

### 2.1. Shannon Entropy

Entropy initially referred to a thermodynamics concept measuring disorder within a system. Shannon introduced it into information theory, leading to the development of “information entropy”, quantifying uncertainty within one or multiple random variables, commonly known as Shannon entropy [19]. For two discrete random variables *X* and *Y* with sets of possible values $\left\{x_{1},x_{2},\ldots ,x_{n}\right\}$ and $\left\{y_{1},y_{2},\ldots ,y_{m}\right\}$, respectively, their joint probability mass function denoted by $p(x_{i},y_{j})$ exists for i=1,2,…,n, j=1,2,…,m. The conditional probability mass function of *X* given *Y* is $p(x_{i}|y_{j})$. Then, the entropy of variable *X* can be defined as

(1) $$H\left(X\right)=-\sum_{i=1}^{n}p(x_{i})\ln p(x_{i}).$$

Extending this to cover the scenario of two random variables *X* and *Y*, the joint entropy H(X,Y) is defined as

(2) $$H\left(X,Y\right)=-\sum_{i=1}^{n}\sum_{j=1}^{m}p(x_{i},y_{j})\ln p(x_{i},y_{j}).$$

Moreover, the conditional entropy H(X|Y) for variables *X* and *Y* is expressed as

(3) $$\begin{array}{cc} H(X|Y) & =\sum_{j=1}^{m}p(y_{j})H(X|Y=y_{j})\\ \; & =-\sum_{i=1}^{n}\sum_{j=1}^{m}p(x_{i},y_{j})\ln p(x_{i}|y_{j}). \end{array}$$

It can be noted that the chain rule holds between H(X), H(X,Y) and H(X|Y), specifically

(4) H(X,Y)=H(X)+H(Y|X).

In particular, when *X* and *Y* are independent,

(5) H(X,Y)=H(X)+H(Y).

Entropy to some extent reflects the complexity of a random variable. Take the example of a random variable *X* that follows a 0–1 distribution. When it degenerates into a single-point distribution, the random variable *X* has only one possible value, indicating no complexity. In this case, H(X)=0 (generally, in information theory, define 0·log0=0 to ensure the continuity of entropy). When *X* follows a uniform distribution between 0 and 1, the values of this random variable become harder to predict, indicating higher complexity. In this scenario, H(X) reaches its maximum value of 1.

### 2.2. General Entropy

On the foundation of Shannon entropy, many generalized forms of entropy have been proposed, often presented in the form with free parameters. Below, we introduce several common generalized entropies. The entropy of order *r* (also known as Rényi entropy) is defined as

(6) $$H_{r}\left(X\right)=\frac{1}{1-r}\ln\left(\sum_{i=1}^{n}{\left(p\left(x_{i}\right)\right)}^{r}\right),$$

where r>0 and r≠1. It is easy to see that $\lim_{r\to 1}H_{r}\left(X\right)=H\left(X\right)$. In other words, H(X) is the limit form of the $H_{r}\left(X\right)$ as r→1.

The entropy of degree *r* (also known as Tsallis entropy) is defined as

(7) $$H^{r}\left(X\right)=\frac{1}{1-r}(\sum_{i=1}^{n}{\left(p\left(x_{i}\right)\right)}^{r}-1),$$

where r>0 and r≠1. It can be easily demonstrated that $\lim_{r\to 1}H^{r}\left(X\right)=H\left(X\right)$. Specifically, when $r=2,H^{2}\left(X\right)$ represents the Gini index, which is a metric describing diversity, i.e.,

(8) $$H^{2}\left(X\right)=1-\sum_{i=1}^{n}{\left(p\left(x_{i}\right)\right)}^{2}.$$

The entropy of type *r* is defined as

(9) H(X)r=1r−1[(∑i=1n(p(xi))1r)r−1],

where r>0 and r≠1. Similarly, as r→1, H(X)r converges to the Shannon entropy H(X), i.e., limr→1H(X)r=H(X). The RS-entropy is defined as

(10) $$H_{r}^{s}\left(X\right)=\frac{1}{(1-r)s}\left[{\left(\sum_{i}^{n}{\left(p\left(x_{i}\right)\right)}^{r}\right)}^{s}-1\right],$$

where r>0, r≠1, and s≠0. It is not difficult to observe that $H^{r}\left(X\right)$ is a special case of $H_{r}^{s}\left(X\right)$ when s=, i.e.,

(11) $$H_{r}^{1}\left(X\right)=\frac{1}{\left(1-r\right)}\left[\sum_{i=1}^{n}{\left(p\left(x_{i}\right)\right)}^{r}-1\right]=H^{r}\left(X\right).$$

When s=α and $r=\frac{1}{\alpha }$, where α>0 and α≠1, $H_{r}^{s}\left(X\right)$ degenerates into H(X)r, i.e.,

(12) Hrs(X)=1(α−1)[(∑i=1n(p(xi))1α)α−1]=H(X)α.

It can be easily proven that $H_{r}\left(X\right)$ is the limit form of $H_{r}^{s}\left(X\right)$ as s→0, i.e., $\lim_{s\to 0}H_{r}^{s}\left(X\right)=H_{r}\left(X\right)$. Similarly, H(X) is the limit form of $H_{r}^{s}\left(X\right)$ as s=1 and r→1, i.e., $\lim_{r\to 1}H_{r}^{1}\left(X\right)=H\left(X\right)$. Combining (1), (6), (7), (9) and (10), we can derive a unified RS-entropy form, denoted as $E_{s}^{r}\left(X\right)$(r>0), specifically as

(13) Ers(X)=Hrs(X),r≠1,s≠0,Hr(X),r≠1,s=0,Hr(X),r≠1,s=1,H(X)r,r≠1,s=1r,H(X),r=1,s=1.

## 3. Classification Algorithm Based on RS-Entropy

In this section, we describe the classification tasks that decision tree algorithms address. We then elucidate the application of information entropy in the algorithmic process and present the steps of our designed classification algorithm based on RS-entropy.

### 3.1. Problem Description

For a given dataset *D*, which contains |D| samples, each data sample is represented by (X,Y). *X* represents the attribute vector $\left(X_{1},X_{2},\ldots ,X_{n}\right)$ for each data sample, where each $X_{i}$ serves as a random variable taking values from their respective finite sets $X_{i}=\left\{x_{i1},x_{i2},\ldots ,x_{in_{i}}\right\}$. *Y* represents the class of the data sample, serving as a random variable, taking values from the class set Y={1,2,…,K}.

The task is to construct a decision tree for classification based on the dataset. In this tree, each node represents a subset of the dataset, and the branching of the tree signifies a division of the dataset associated with that node. Nodes between different levels are connected by branches, indicating the splitting criteria used from one level to the next. To stop the growth of the tree, a criterion is set to determine when no further branching should occur. Nodes that do not branch further are called leaves, and they return the class with the most samples within that leaf as the predicted class.

For a new data example $\left(\tilde{X},\tilde{Y}\right)$, we can start from the root node of the constructed tree and, based on the feature values of the sample, follow the branches sequentially until you reach a leaf node in the decision tree. The class associated with the leaf node is the model’s predicted classification result $\hat{Y}$, thus completing the classification task.

### 3.2. Split Criterion

Different splitting and stopping criteria can lead to variations in the constructed tree, thereby influencing the predicted categories. Stopping criteria are typically used to limit the complexity of the tree, control computational resources, and prevent overfitting. These criteria often involve restrictions related to the overall depth of the tree, the number of samples in leaf nodes, or the complexity of samples in leaf nodes [20].

This article primarily focuses on splitting criteria. The objective of splitting can be summarized as finding the most suitable attribute and attribute value to partition the dataset in such a way that the complexity of categories in the resulting partitions is minimized as much as possible. Various methods are available to characterize complexity, such as Shannon entropy and Gini index. The entropy of degree *r* exhibits favorable properties and achieves a unification of Shannon entropy and the Gini index.

#### 3.2.1. The Two-Term Information Method

The two-term information method in decision trees was introduced by Wang. This measure represents a novel splitting criterion for decision trees. It not only computes the complexity of categories within the subsets generated by splitting on a particular attribute but also takes into account the complexity of attributes within the same category within those subsets. The sum of these two complexities is used as the splitting criterion. Compared to traditional methods, this construction approach is less greedy. While it may not guarantee that each individual split is optimal according to traditional standards, it achieves an overall better outcome. We will use the dataset from Table 1 as an example to illustrate this approach.

To facilitate the description, we establish the following notations:

- The splitting point is denoted as $X_{i}\left(a\right)$, and it is used to partition the dataset *D* into two subdatasets, represented as $D_{1}=\left\{\left(X,Y\right)|X_{i}\leq a\right\}$ and $D_{2}=\left\{\left(X,Y\right)|X_{i}>a\right\}$.
- The complexity of category *Y* in dataset *D* is denoted as H(Y|D), which calculates the empirical entropy of category *Y* in dataset *D*. For ease of calculation in the example, we use empirical Shannon entropy to quantify the complexity.
- The complexity of the attribute $X_{i}$ with respect to the category *Y* in the dataset *D* is denoted as $H(X_{i}|D,Y)$. Its calculation formula is
  (14) $$H\left(X_{i}|D,Y\right)=\sum_{y}\frac{|D_{Y=y}|}{\left|D\right|}H\left(X_{i}|D_{Y=y}\right),$$
  where $D_{Y=y}=\left\{\left(X,Y\right)|Y=y\right\}$.
- The variance of the attribute $X_{i}$ in dataset *D* is denoted as $V(X_{i}|D)$, and its calculation formula is
  (15) $$V\left(X_{i}|D\right)=\frac{1}{n_{i}}\sum_{j=1}^{n_{i}}(x_{ij}-\frac{1}{n_{i}}\sum_{j=1}^{n_{i}}x_{ij}{)}^{2}.$$
- The complexity of splitting point $X_{i}\left(a\right)$ is denoted as $H(X_{i}\left(a\right))$. It represents the weighted sum of the complexities of subsets $D_{1}$ and $D_{2}$ after partitioning dataset *D* with respect to the sample proportions.

It is straightforward to calculate that when using the splitting point $X_{1}\left(3.5\right)$ to divide dataset *D* into two subsets $D_{1}$ and $D_{2}$, where $D_{1}=\left\{D|X_{1}\leq 3.5\right\}$ contains data examples with index 1, 2, and 3. The complexity with respect to category *Y* for the subsets is $H(Y|D_{1})=0$. Similarly, we have $H(Y|D_{2})=0$. By weighting the complexities of the two subsets according to the sample proportions, we can calculate the category complexity under the splitting point $X_{1}\left(3.5\right)$ as follows:

(16) $$H\left(X_{1}\left(3.5\right)\right)=\frac{3}{6}H\left(Y|D_{1}\right)+\frac{3}{6}H\left(Y|D_{2}\right)=0.$$

Similarly, we can calculate the category complexity under splitting point $X_{2}\left(1.5\right)$:

(17) $$H\left(X_{2}\left(1.5\right)\right)=\frac{3}{6}H\left(Y|D_{1}\right)+\frac{3}{6}H\left(Y|D_{2}\right)=0.$$

We should seek the splitting point that minimizes the complexity of categories after splitting. The results from Equations (16) and (17) indicate that both splitting points $X_{1}\left(3.5\right)$ and $X_{2}\left(1.5\right)$ have the same effect, resulting in a category complexity of 0 after splitting. However, it is evident that splitting point $X_{2}\left(1.5\right)$ is superior to $X_{1}\left(3.5\right)$ because attribute $X_{2}$ appears to have a stronger correlation with *Y*, indicating that the values of $X_{2}$ may directly influence category *Y*. In essence, this method of determining splitting points based on the complexity of categories within the resulting subsets has limitations as it overlooks the relationship between attributes and categories during the splitting process.

The two-term information methods have improved the way splitting point complexity is calculated, enhancing the identification capability of splitting points. In characterizing the complexity of subset $D_{1}$, this method calculates the sum of the set’s category complexity $H(Y|D_{1})$ and attribute complexity $H(X_{i}|D_{1},Y)$, differently from the traditional splitting approach that solely considers category complexity while ignoring attribute complexity. Then we can calculate the category complexity under splitting point $X_{1}\left(3.5\right)$:

(18) $$\begin{array}{cc} H(X_{1}\left(3.5\right)) & =\frac{3}{6}\left[H\left(Y|D_{1}\right)+H\left(X_{1}|D_{1},Y\right)\right]+\frac{3}{6}\left[H\left(Y|D_{2}\right)+H\left(X_{1}|D_{2},Y\right)\right]\\ \; & =\ln3. \end{array}$$

Similarly, we have the category complexity under splitting point $X_{2}\left(1.5\right)$:

(19) $$\begin{array}{cc} H(X_{2}\left(1.5\right)) & =\frac{3}{6}\left[H\left(Y|D_{1}\right)+H\left(X_{1}|D_{1},Y\right)\right]+\frac{3}{6}\left[H\left(Y|D_{2}\right)+H\left(X_{1}|D_{2},Y\right)\right]\\ \; & =0. \end{array}$$

**Figure 1:**
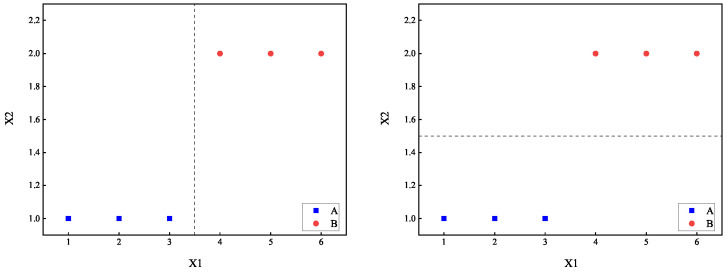
Comparison of the splitting effects of $X_{1}\left(3.5\right)$ and $X_{2}\left(1.5\right)$.

Comparing (18) and (19), since $H\left(X_{2}\left(1.5\right)\right)\leq H\left(X_{1}\left(3.5\right)\right)$, this supports our choice to split the dataset using splitting point $X_{2}\left(1.5\right)$. The splitting effect of $X_{1}\left(3.5\right)$ and $X_{2}\left(1.5\right)$ are illustrated in the left and right sub-figures of Figure 1.

The two-term information method evidently performs better in attribute selection compared to traditional methods. The concept of this method can be understood as adding a penalty term $H(X_{i}|D,Y)$ on top of the traditional information measures. A good splitting point should not only effectively differentiate categories but also have a strong association between the split attribute and categories. This type of classification method is ideal as it combines both classification accuracy and interpretability. In other words, after splitting based on attribute $X_{i}$, if the values of $X_{i}$ in the resulting subset are too dispersed, it implies that the relationship between this attribute and categories is not strong enough. Clearly, such a splitting point is not optimal, which will be reflected in the relatively larger value of the penalty term $H(X_{i}|D,Y)$. This leads to a higher overall $H(X_{i}\left(x_{ij}\right))$, thereby avoiding the selection of that splitting point as much as possible.

This penalty term has practical significance, as it means that the splitting criterion takes into account the degree of feature confusion. Especially in the random forest algorithm, we need to evaluate the importance of features based on the splitting method of each decision tree. Compared with the traditional feature importance calculated only based on the category Y, this penalty-term-included approach is bound to make the feature ranking more scientific. However, this paper does not elaborate further on this aspect.

#### 3.2.2. Improved Two-Term Information Method

The two-term information method mentioned above still appears to have potential areas for improvement, which can be summarized in the following two aspects:

- The types of attribute $X_{i}$ have not been taken into consideration. In reality, $X_{i}$ can be either a numerical or a categorical attribute, and the computed penalty term $H(X_{i}|D,Y)$ should differ accordingly.
- The penalty term should be more adaptable to better suit different datasets. Introducing a penalty coefficient λ(λ∈[0,1]) before $H(X_{i}|D,Y)$, controlling the weight of $H(X_{i}|D,Y)$ by adjusting λ, can enhance its flexibility.

For example, consider the dataset in Table 2.

Using the two-term information method from Section 3.2.1, we calculate $H\left(X_{1}\left(3.5\right)\right)=H\left(X_{2}\left(1.6\right)\right)=\ln3$, indicating that the splitting points $X_{1}\left(3.5\right)$ and $X_{2}\left(1.6\right)$ are equally optimal. However, it is noticeable that $X_{2}\left(1.6\right)$ should be more suitable as a splitting point. This suggests that the calculation of $H(X_{i}|D,Y)$ may not be suitable for numerical attributes $X_{i}$. For such attributes, we define the conditional variance $V(X_{i}|D,Y)$ to characterize their complexity. Similar to (14), it is defined as

(20) $$V\left(X_{i}|D,Y\right)=\sum_{y}\frac{|D_{Y=y}|}{\left|D\right|}V\left(X_{i}|D_{Y=y}\right).$$

Therefore, the improved computation of the two-term information method can be provided by Equations (21)–(23).

(21) $$H\left(X_{i}\left(a\right)\right)=\left\{\begin{array}{cc} \frac{|D_{1}|}{\left|D\right|}H_{1C}+\frac{|D_{2}|}{\left|D\right|}H_{2C}, & X_{i}\in C\left(X\right),\\ \frac{|D_{1}|}{\left|D\right|}H_{1D}+\frac{|D_{2}|}{\left|D\right|}H_{2D}, & X_{i}\in D\left(X\right), \end{array}\right.$$

where C(X) and D(X) denote the sets of numerical and categorical attributes in the dataset and $H_{jC}$ and $H_{jD}$ are defined as follows:

(22) $$H_{jC}=H\left(Y|D_{j}\right)+\lambda H\left(X_{i}|D_{j},Y\right),\;j=1,2,\;\lambda \in \left[0,1\right].$$

(23) $$H_{jD}=H\left(Y|D_{j}\right)+\mu V\left(X_{i}|D_{j},Y\right),\;j=1,2,\;\mu \in \left[0,1\right].$$

For example, considering the dataset in Table 2, let us assume that both attributes, $X_{1}$ and $X_{2}$, are continuous. Given λ=0.5, the calculated improved $H(X_{1}\left(3.5\right))$ is as follows:

(24) $$\begin{array}{cc} H(X_{1}\left(3.5\right)) & =\frac{3}{6}\left[H\left(Y|D_{1}\right)+\frac{1}{2}V\left(X_{1}|D_{1},Y\right)\right]+\frac{3}{6}\left[H\left(Y|D_{2}\right)+\frac{1}{2}V\left(X_{1}|D_{2},Y\right)\right]\\ \; & =\frac{1}{6}. \end{array}$$

Similarly, $H(X_{2}\left(1.6\right))$ can be computed as well:

(25) $$\begin{array}{cc} H(X_{2}\left(1.6\right)) & =\frac{3}{6}\left[H\left(Y|D_{1}\right)+\frac{1}{2}V\left(X_{1}|D_{1},Y\right)\right]+\frac{3}{6}\left[H\left(Y|D_{2}\right)+\frac{1}{2}V\left(X_{1}|D_{2},Y\right)\right]\\ \; & =\frac{1}{600}. \end{array}$$

**Figure 2:**
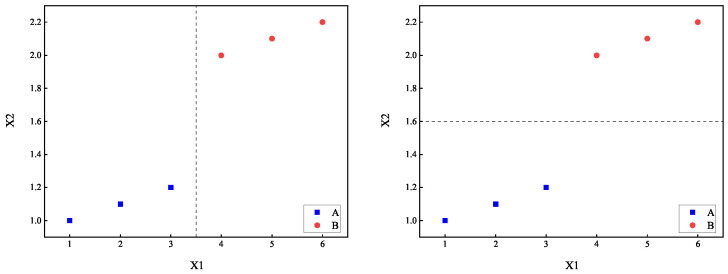
Comparison of the splitting effects of $X_{1}\left(3.5\right)$ and $X_{2}\left(1.6\right)$.

Therefore, $H\left(X_{2}\left(1.6\right)\right)\leq H\left(X_{1}\left(3.5\right)\right)$, indicating that selecting $X_{2}\left(1.6\right)$ as the splitting point results in subsets with both attributes and categories having smaller complexities, making it superior to $X_{1}\left(3.5\right)$ as a splitting point. Figure 2 illustrates the comparison of the dataset partitioning effects between $X_{1}\left(3.5\right)$ and $X_{2}\left(1.6\right)$.

### 3.3. Evaluation of Feature Importance

The two-term information method differs from traditional measurement approaches based on entropy or Gini index in that it incorporates the degree of feature dispersion under the constraint of sample categories. Subsets partitioned using this measurement method not only maximize the distinction between sample categories but also prioritize features with the minimum possible variance as the target for node splitting. Consequently, this method naturally revises the conventional evaluation criteria for feature importance—a modification of great significance in the random forest algorithm.

For two features equally capable of fully distinguishing sample categories, their importance scores are identical under traditional metrics. However, after integrating the measurement of feature variance, the feature with a smaller variance will yield a higher importance score. More specifically, we denote the feature importance measurement as VIM and the Gini index as GI. Assuming that there are *c* features $X_{1},...,X_{c}$, the importance score of feature $X_{1}$ is denoted as $VIM_{1}^{\mu }$, which represents the average reduction in node splitting impurity attributed to feature $X_{1}$ across all decision trees within the random forest.

The calculation formula for the Gini index of node *m* is as follows:

(26) $$GI_{m}=1-\sum_{k=1}^{\left|K\right|}p_{mk}^{2},$$

where |K| denotes the number of categories and $p_{m}k$ represents the proportion of category *k* in node *m*. Similarly to Equation (23), assuming that $X_{1}$ is a continuous feature, the two-term Gini index for $X_{1}$ is defined as follows:

(27) $$GI_{m}^{\mu }=1-\sum_{k=1}^{\left|K\right|}p_{mk}^{2}+\mu V\left(X_{1}|D_{m},Y\right),$$

The importance of feature $X_{1}$ at node *m* is defined as the difference between the Gini index $GI_{m}$ of node *m* and the variance-corrected Gini indices $GI_{l}^{\mu }$ and $GI_{r}^{\mu }$ of the two child nodes (denoted as *l* and *r*) formed after splitting [21]. Specifically, it is expressed as follows:

(28) $$\begin{array}{cc} VIM_{1,m} & =GI_{m}-GI_{l}^{\mu }-GI_{r}^{\mu }\\ \; & =-1-\sum_{k=1}^{\left|K\right|}p_{mk}^{2}+\sum_{k=1}^{\left|K\right|}p_{lk}^{2}+\sum_{k=1}^{\left|K\right|}p_{rk}^{2}-\mu V\left(X_{1}|D_{l},Y\right)-\mu V\left(X_{1}|D_{r},Y\right) \end{array}$$

Assuming there are *n* trees in the random forest, the normalized importance score of feature $X_{1}$ is given by

(29) $$VIM_{1}=\frac{\sum_{m=1}^{n}VIM_{1,m}}{\sum_{i=1}^{c}\sum_{m=1}^{n}VIM_{i,m}}.$$

When μ=0, this calculation method for $VIM_{1}$ degenerates into the feature importance score of a random forest constructed using the traditional Gini index as the splitting criterion. The $VIM_{1,m}$ calculated with the improved splitting criterion includes $\mu V(X_{1}|D_{l},Y)$ and $\mu V(X_{1}|D_{r},Y)$; specifically, the greater the variance of feature $X_{1}$ in the split child subsets, the lower the resulting importance score. Considering the inconsistent units among different features, it is necessary to normalize the features in advance and attach a penalty coefficient μ to the variance. In this way, features with high calculated scores will simultaneously possess the ability to distinguish category *Y* and a low variance of their own.

### 3.4. RSEIM Algorithm

In Section 2.2, we introduced the concept of rs‐entropy, and in Section 3.2.2, we proposed an improved two-term information method. Now, we can integrate these two by replacing Shannon entropy with rs‐entropy in Section 3.2. This amalgamation constitutes the RSEIM algorithm proposed in this paper. Initially, we present the symbols and equations employed in this algorithm:

(30) $$E_{r}^{s}\left(X_{i}\left(a\right)\right)=\left\{\begin{array}{cc} \frac{|D_{1}|}{\left|D\right|}E_{r,1C}^{s}+\frac{|D_{2}|}{\left|D\right|}E_{r,2C}^{s}, & X_{i}\in C\left(X\right),\\ \frac{|D_{1}|}{\left|D\right|}E_{r,1C}^{s}+\frac{|D_{2}|}{\left|D\right|}E_{r,2C}^{s}, & X_{i}\in D\left(X\right). \end{array}\right.$$

(31) $$E_{r,jC}^{s}=E_{r}^{s}\left(Y|D_{j}\right)+\lambda E_{r}^{s}\left(X_{i}|D_{j},Y\right),\;j=1,2,\;\lambda \in \left[0,1\right],$$

(32) $$E_{r,jD}^{s}=E_{r}^{s}\left(Y|D_{j}\right)+\mu E_{r}^{s}\left(X_{i}|D_{j},Y\right),\;j=1,2,\;\mu \in \left[0,1\right].$$

The specific steps are outlined by the pseudocode in Algorithm 1.

**Algorithm 1:** RSEIM algorithm
**Input**:
Data *D*, Attribute *X*, Class *Y***Output**:
A decision tree1:  **while** not satisfying the stop condition **do**2:     Initialize $E_{min}\leftarrow$ Inf3:     **for** each attribute $X_{i}$ **do**4:       **for** each cutting point $X_{i}\left(a_{ij}\right)$ **do**5:          **if** $X_{i}\in C\left(X\right)$ **then**6:              $D_{1}\leftarrow \left\{D|X_{i}\leq X_{i}\left(a_{ij}\right)\right\}$7:              $D_{2}\leftarrow \left\{D|X_{i}>X_{i}\left(a_{ij}\right)\right\}$8:          **else**9:              $D_{1}\leftarrow \left\{D|X_{i}=X_{i}\left(a_{ij}\right)\right\}$10:            $D_{2}\leftarrow \left\{D|X_{i}\neq X_{i}\left(a_{ij}\right)\right\}$11:        **end if**12:        Compute $E_{r}^{s}\left(X_{i}\left(a_{ij}\right)\right)$ according to (30)13:     **end for**14:     **if** $E_{r}^{s}\left(X_{i}\left(a_{ij}\right)\right)$ < $E_{min}$ **then**15:        a←$a_{ij}$16:        X←$X_{i}$17:        $E_{min}\leftarrow$$E_{r}^{s}\left(X_{i}\left(a_{ij}\right)\right)$18:     **end if**19:   **end for**20:   Grow the tree using *X* and *a*, partitioning the data via binary split.21:   Go to the beginning for $D_{1}$ and $D_{2}$22:   // Recursively repeat the procedure to grow a tree23:
**end while**
24:**Return:** A decision tree25:// Tree is built by nodes from the root to the leaf


It is evident that besides the parameters included in the stopping criteria, the algorithm also comprises four free parameters: *r* and *s* from the rs‐entropy $E_{r}^{s}\left(X\right)$, along with penalty coefficients λ and μ from the improved two-term information method. Particularly, when λ=0 and μ=0, the algorithm degenerates into a decision tree algorithm based on rs‐entropy without penalty terms. In this paper, we refer to this algorithm as RSE. Additionally, by setting the condition s=1 on the RSE algorithm, it degrades to a decision tree algorithm based on Tsallis entropy, termed TE. Setting r=1 on the TE algorithm leads to its degradation into a decision tree algorithm based on Shannon entropy, denoted as SE in this paper. If the condition is altered to r=2, it degrades into a decision tree algorithm based on Gini coefficient, labeled as GN in this paper.

The time complexity of a single split in RSEIM depends on the number of features *m* and the number of samples *n*. For continuous features, the time complexity of model training is O(m·n·logn), while for discrete features, it is O(m·n), which is comparable to that of traditional decision tree models without hyperparameters [17]. In fact, the construction process of RSEIM can be simply regarded as building several decision trees with different parameters and selecting the one with the best performance. The number of such trees depends on the density of parameter pairs selected in the space. Compared with a single tree, the difference in complexity only lies in a larger constant factor, and the increase in the number of features and samples will not lead to higher time complexity. However, the storage of multiple parameters will result in higher space complexity, which depends on the spatial density of the selected parameters.

## 4. Experiment and Evaluation

To compare the performance differences among different algorithms, we conducted experiments on multiple datasets. We primarily used prediction accuracy as the evaluation criterion for the test set, with the confusion matrix serving as a supplementary reference. Regarding parameter selection, we traversed through various parameter combinations for two parameters to find the optimal pair. For scenarios involving more parameters, we introduced optimization algorithms to search for the best parameter sets.

### 4.1. Experimental Data

The 8 datasets used in the experiments were sourced from the UCI Machine Learning Repository. These datasets encompass varying sample sizes, numbers of attributes, class counts, and types of attributes. Detailed information about the datasets is provided in Table 3.

### 4.2. Experimental Result

We evaluated two algorithms, RSE with two parameters and RSEIM with four parameters, respectively. This evaluation aimed to assess whether incorporating a more general segmentation criterion and the introduction of penalty terms would significantly enhance the model’s performance. For the evaluation of algorithm performance, this paper uses 30% of the data as the test set to obtain the model accuracy on the test set, and adopts 10-fold cross-validation to acquire the average accuracy on the training set [22]. Furthermore, we aggregated all prediction results and true outcomes on the test sets into a confusion matrix.

#### 4.2.1. Experimental Results of RSE Algorithm

The algorithm replaces Shannon entropy and Gini index with the rs-entropy, introducing additional free parameters *r* and *s*. The experiment confines parameters *r* and *s* within the range of 0 to 10, with a step size of 0.1, exploring all parameter pairs. In total, 30% of the data is used as the test set to calculate the model accuracy on the test set. The results are then visualized in the form of a heatmap to intuitively present the experimental outcomes.

**Figure 3:**
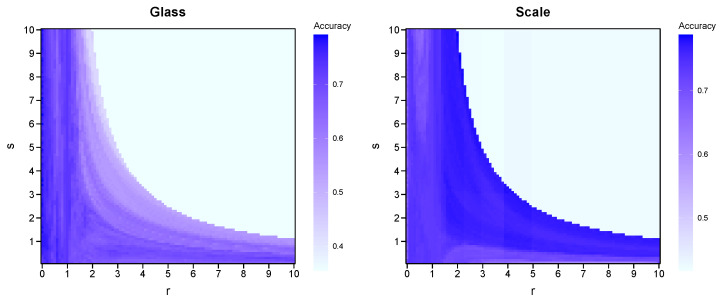
Accuracy of RSE algorithm for Glass dataset and Scale dataset.

Figure 3 displays the heatmaps generated from the Glass dataset and the Scale dataset. In the heatmap of the Glass dataset, the dark regions concentrate in the banded area where r∈(0,1]. The effectiveness of the SE algorithm and GN algorithm is reflected by the depth of color at points (1,1) and (2,1), respectively. It is evident that the SE algorithm performs better on this dataset. When selecting parameters r=0.5 and s=1.8, the RSE algorithm achieves the highest accuracy of 74.89%. For r=1 and s=1, the SE algorithm’s accuracy is 73.35%. With r=2 and s=1, the GN algorithm’s accuracy is 68.33%. In the Scale dataset, the dark regions correspond to higher parameter values. However, in contrast, both the SE and GN algorithms do not perform significantly well. When selecting parameters r=2 and s=8.8, the RSE algorithm achieves the maximum accuracy of 75.49%. For r=1 and s=1, the SE algorithm’s accuracy is 73.23%. With r=2 and s=1, the GN algorithm’s accuracy is 72.61%. Therefore, the parameter pairs that yield the highest accuracy differ across different datasets. This implies the necessity of introducing free parameters *r* and *s*, as their adjustability enhances the algorithm’s adaptability to various datasets. Appropriate free parameters *r* and *s* have the potential to significantly improve the model accuracy compared to traditional SE and GN algorithms.

#### 4.2.2. Experimental Results of RSEIM Algorithm

**Figure 4:**
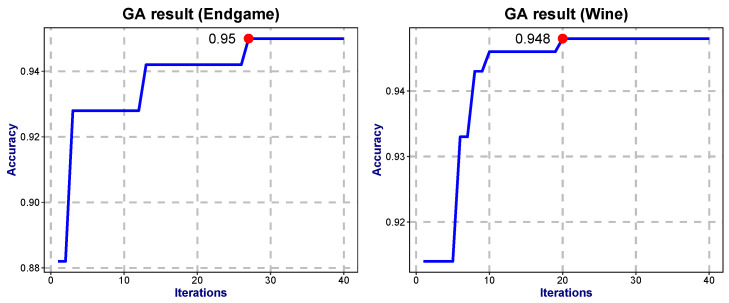
Evolutionary diagram of GA for Endgame dataset and Wine dataset.

The RSEIM algorithm introduces at least one additional free parameter compared to the RSE algorithm. Parameters are confined within the following ranges: r∈(0,10], s∈(0,10], λ∈[0,1], μ∈[0,1]. The objective function is the average accuracy under ten-fold cross-validation. Genetic algorithms were employed to optimize these parameters to maximize the average accuracy [23]. We set the necessary parameters of the genetic algorithm as follows: the length of the independent variable is 10, the population size is 40, the maximum number of iterations is 40, the offspring proportion is 0.6, the mutation probability is 0.05. Figure 4 illustrates the iterative evolution for the Endgame and Wine datasets, while Table 4 presents the results of parameter optimization.

The results indicate that after 40 iterations of evolutionary optimization, the accuracy generally increased by 2–4 percentage points. Specifically, the predicted accuracies for the Glass dataset and Scale dataset reached 74.94% and 75.63%, respectively. Compared with the maximum accuracies obtained via exhaustive search in the RSE algorithm (74.89% and 75.49%, respectively), the difference is minimal—within ±1%. Furthermore, the computational complexity of the genetic algorithm is significantly lower than that of exhaustive search.

The optimization process reveals that different parameter selections exert an impact on the actual performance of the model, which demonstrates the necessity of parameter optimization or presetting. From the perspective of parameter presetting, in the experiments, parameters *r* and *s* tend to be selected as constants greater than 1, parameter μ tends to be a constant less than 0.1, while the selection of parameter λ is more difficult to determine. This may be associated with the complexity of discrete features. From the perspective of parameter optimization, compared with grid search, the genetic algorithm employed in this experiment reduces the number of computations while achieving relatively favorable model performance; this result will be analyzed in detail in the next section. Certainly, there exist various optimization algorithms. Since this paper primarily focuses on discussing the significance and necessity of parameters, no additional parameter optimization experiments have been conducted.

#### 4.2.3. Ablation Study Analysis

**Figure 5:**
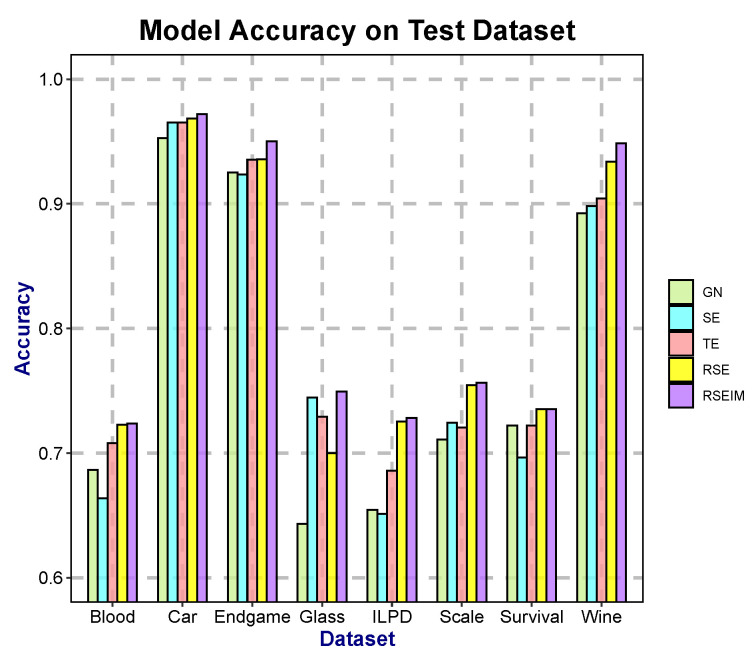
Comparison of the accuracy of different algorithms.

**Figure 6:**
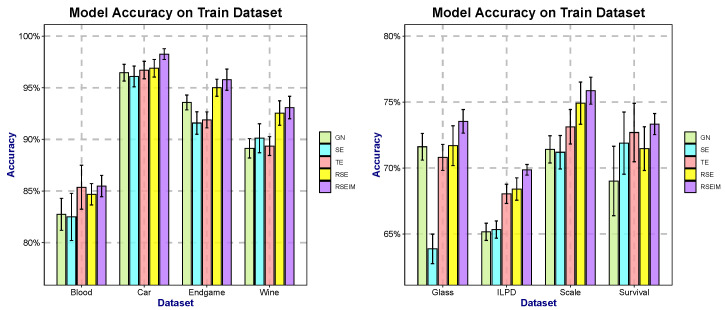
Comparison of the results of different algorithms on selected datasets.

To evaluate the contribution of the newly introduced parameters *r*, *s*, λ, and μ to the model performance, five algorithms—SE, GN, TE, RSE, and RSEIM—were compared. Specifically, the TE algorithm incorporates component *r* into the original SE and GN algorithms; the RSE algorithm further introduces component *s* based on TE; and the RSEIM algorithm additionally integrates components λ and μ on the basis of RSE. To ensure a fair comparison among these different methods, we applied identical stopping conditions and set a random seed to guarantee consistency between the training and test sets. As the theoretical support for algorithm optimization and enhancement has been established in Section 2 and Section 3, this section presents experimental comparisons of the average accuracy among different algorithms, to demonstrate the impact of the introduced components on the model. We present the accuracy of each model on the test set, respectively, as shown in Table 5 and Figure 5. The test data accounts for 30% of the total dataset, which allows for an intuitive comparison of model effectiveness. Additionally, we adopt 10-fold cross-validation to demonstrate the performance of the models on the test set during the training process, as illustrated in Figure 6.

The experimental results indicate that on the majority of datasets, both RSE and RSEIM algorithms demonstrate notable performance on test data. On the Scale dataset, their predictive accuracy improves by 2–3% compared to the traditional SE and GN algorithms. On the Car dataset, the predictive accuracy increases by 1–2%, while on the Survival dataset, the accuracy enhancement ranges between 1–2%.

We separately obtained two types of results: the test results using 30% of the data as the test set (Figure 5) and the training results from 10-fold cross-validation (Figure 6). Based on these, we comprehensively compared the differences between algorithms integrated with different components. Both RSE and RSEIM algorithms achieved better performance on both the test set and the training set. Furthermore, the acquisition of test performance and training performance is independent, allowing us to rule out the possibility of overfitting and data leakage. Among the training results of the 10-fold cross-validation, the RSEIM algorithm not only achieved the best average accuracy but also had a lower standard deviation. This indicates that the newly introduced components have enhanced the stability of the model, making it applicable to more diverse training data.

**Figure 7:**
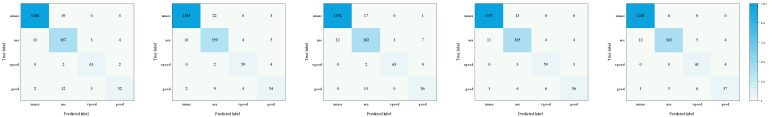
Confusion matrices of SE, GN, TE, RSE, and RSEIM algorithms on Car dataset.

**Figure 8:**
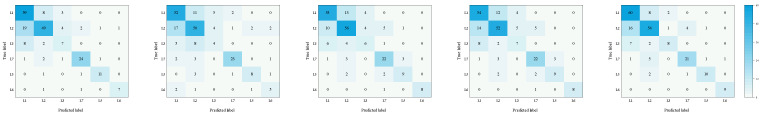
Confusion matrices of SE, GN, TE, RSE, and RSEIM algorithms on Glass dataset.

**Figure 9:**
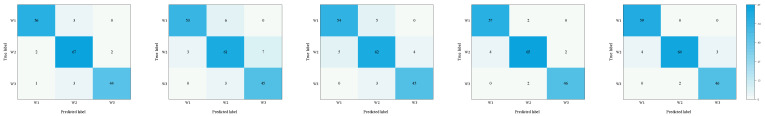
Confusion matrices of SE, GN, TE, RSE, and RSEIM algorithms on Wine dataset.

In addition to overall accuracy, we also generated confusion matrices for five classification algorithms on selected datasets (Figure 7, Figure 8 and Figure 9). The confusion matrix is a table used in machine learning to depict the analysis of a classification model’s predictions. It summarizes experimental data based on true classes and predicted classes by the classification models, presented in matrix form. This matrix provides a clear visualization of performance differences among different algorithms.

From the confusion matrices, it is evident that distinguishing clear superiority between the SE and GN algorithms is challenging. However, the TE algorithm combines the strengths of the former two, achieving better results by adjusting parameters. The confusion matrix demonstrates its more accurate predictions in most categories compared to the traditional algorithms. On the other hand, the RSE and RSEIM algorithms, by introducing more free parameters and decision penalty terms, show further performance improvement. In the Car dataset, for predictions in the ’unacc’ category, the SE and GN algorithms accurately predicted 1188 and 1185 instances, respectively, whereas the RSE and RSEIM algorithms achieved 1197 and 1240 accurate predictions, respectively. For predictions in the ’good’ category, the SE and GN algorithms accurately predicted 52 and 54 instances, respectively, while the RSE and RSEIM algorithms achieved 56 and 57 accurate predictions, respectively.

#### 4.2.4. Non-Parametric Test

To quantitatively test the significance of differences among the SE, GN, TE, RSE, and RSEIM algorithms, we consider using the Nemenyi non-parametric test to evaluate the performance of multiple models [24]. The Nemenyi test is suitable for comparing multiple groups of experiments involving *k* algorithms on *N* datasets. By comparing the ranking ranks of specific indicators and combining the Critical Difference threshold (hereinafter referred to as CD), it can identify models with significantly superior performance. The threshold CD is calculated as

(33) $$CD=q_{\alpha }\sqrt{\frac{k(k+1)}{6N}},$$

where $q_{\alpha }$ is a constant determined by the number of algorithms *k*, the number of datasets *N*, and the significance level α. Here, we compared the accuracy rankings of the five algorithms (SE, GN, TE, RSE, RSEIM) on four datasets (Car, Glass, Scale, Wine). The average accuracy was calculated using 30% of the data as the test set, and this experiment was conducted independently ten times. Thus, k=5, N=10, and α=0.05. From the table, $q_{\alpha }=2.1$, and the calculated critical difference value is 1.48. That is, algorithms with an average rank difference in accuracy greater than 1.48 can be considered to have significant differences.

**Figure 10:**
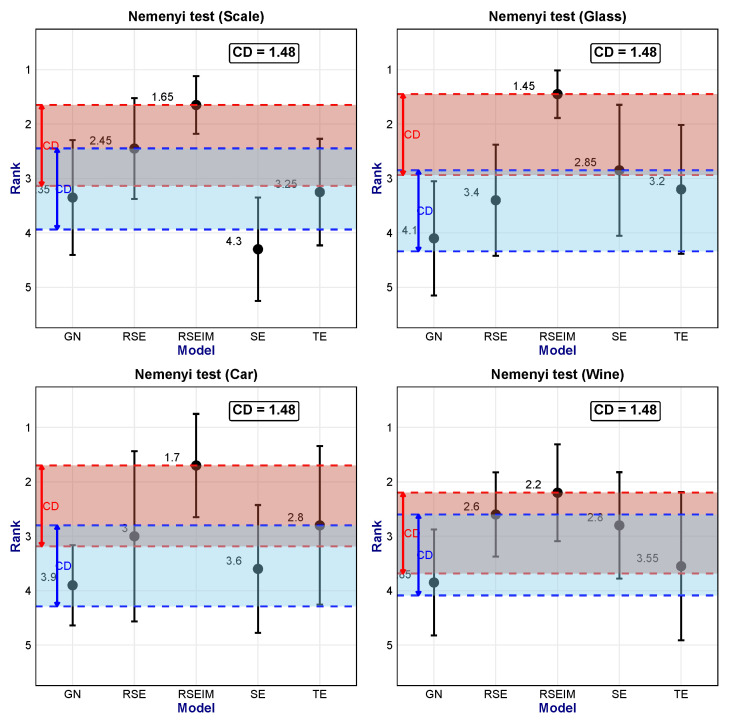
Nemenyi test of result in Scale, Glass, Car and Wine dataset.

On the above-mentioned four datasets, RSEIM achieved the highest average rank in accuracy, as showwn in Figure 10. It was significantly superior to the traditional GN and SE algorithms on the Car, Glass, and Scale datasets, and significantly superior to the GN algorithm on the Wine dataset, while the difference from SE did not pass the significance test (p<0.05). In addition, experiments on the four datasets showed that although the traditional GN and SE algorithms use different splitting criteria, no significant difference was found in terms of accuracy on the test set. Other optimized models, TE and RSE, although their average ranks were improved compared with traditional models, generally failed to pass the Nemenyi test, and the improvement effect was not significant (p>0.05).

#### 4.2.5. Comparison of Trees Complexity

We have theoretically and experimentally validated that the RSE and RSEIM algorithms are significantly superior to traditional decision tree algorithms and single-parameter decision tree algorithms. However, during the experimental process, we predefined stopping conditions, including the maximum depth of trees and the minimum size of leaves. In fact, these stopping conditions were set based on our experience, but to a large extent, they restricted the complexity of the trees, especially the maximum depth, which directly determines the upper limit of tree complexity. An effective decision tree should not only possess higher accuracy but also exhibit lower complexity. In other words, we aim for trees with the smallest depth possible while maintaining high accuracy. Therefore, we conducted experiments on the five aforementioned classification algorithms under different tree depths to explore the correlation between maximum depth and classification accuracy.

**Figure 11:**
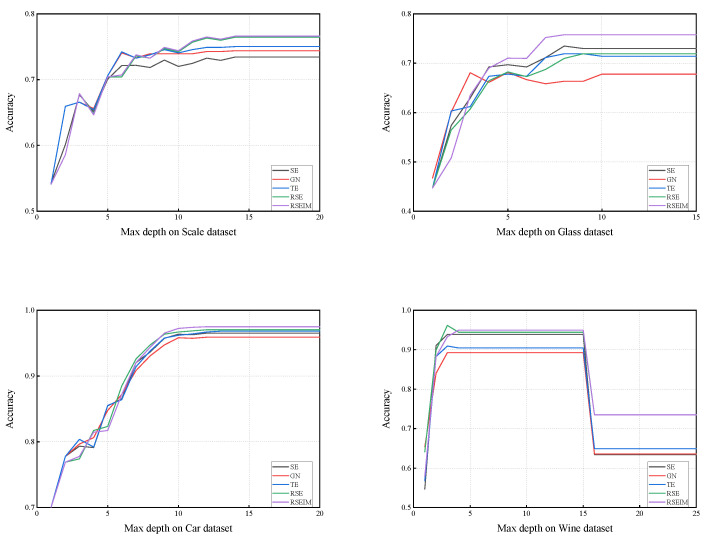
Max depth and accuracy on selected datasets.

The graphs (Figure 11) illustrate the prediction accuracy of different algorithms concerning the maximum depth on the Scale, Glass, Car, and Wine datasets. In pursuit of the highest accuracy with minimal tree depth, the optimal depth is identified as the peak point located towards the left in the line graphs. For instance, on the Scale dataset, the optimal depths for all five algorithms are around 10. On the Glass dataset, optimal depths are around 8. On the Wine dataset, the optimal depths for all five algorithms range between 4–15. Notably, beyond a depth of 15, a decrease in accuracy due to overfitting is observed. However, even in such cases, the RSEIM algorithm maintains higher accuracy compared to other algorithms. Experimental results across most datasets show that the optimal depth does not significantly differ among different algorithms. This suggests that both RSE and RSEIM algorithms not only improve accuracy but also do not increase tree complexity. This, in turn, indicates their superior performance compared to traditional decision trees.

#### 4.2.6. Noise Analysis Based on Artificial Data

In Section 3.3, we discussed the optimization of the improved decision tree algorithm in terms of feature recognition capability. In brief, the two stage method enables the decision tree to tend toward selecting features with low variance. In fact, features with low variance not only hold greater practical significance but also imply less noise interference on such features. When features exhibit similar category recognition capabilities, it is reasonable to assign higher importance to these low-variance features. In this section, we use artificially generated datasets to verify the differences in feature recognition capabilities among algorithms when the data is subject to noise interference.

We set up two types of data, Class *A* and Class *B*, both containing two features (X and Y), each following a normal distribution, $X_{A}∼N\left(2,0.6\right)$, $Y_{A}∼N\left(4,0.3\right)$, $X_{B}∼N\left(6,0.6\right)$, $Y_{B}∼N\left(8,0.3\right)$. Obviously, decision tree algorithms can easily distinguish between samples of Class *A* and Class *B* using feature nodes X=4 and Y=6. Traditional decision trees assign the same level of importance to Features *X* and *Y* (calculated via Equation (26)). In contrast, the two-stage optimization evaluates features using Equation (27) and thus selects Feature *X* (with smaller variance) as the preferred feature. Gaussian noise of varying intensities was added to Feature *Y*. We evaluated the VIM assigned to Features *X* and *Y* by different algorithms after one splitting iteration, in accordance with Equation (29). Table 6 presents the parameter settings used in the algorithms, while Table 7 shows the VIM values under different noise intensities.

**Figure 12:**
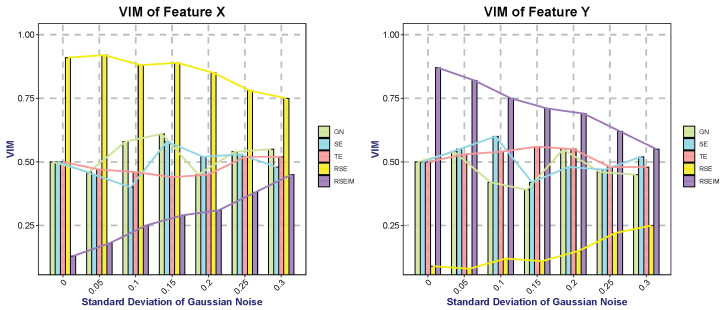
Comparison of the results about different algorithms on selected datasets.

Figure 12 illustrates the VIM with noise intensity. The GN, SE, and TE algorithms do not incorporate feature complexity when calculating VIM. Consequently, they cannot distinguish the superiority between the two features when the feature means are sufficient for category differentiation. As the standard deviation of noise added to Feature *Y* increases from 0 to 0.3, its VIM values remain within the range of 0.4–0.6. For the RSE algorithm, Feature *Y* exhibits a lower VIM under low-noise conditions; however, as stronger noise is added to Feature *Y*, its VIM gradually increases. This trend is opposite to that of the RSEIM algorithm. The reason for this lies in the fact that RSEIM incorporates a weight for feature complexity when calculating VIM, which reduces the sensitivity of VIM to feature complexity. When the noise intensity approaches the variance of the data itself, the VIM values of Features *X* and *Y* calculated by RSEIM are relatively close (0.45 and 0.55, respectively), and this result is more consistent with human intuition.

## 5. Conclusions

In this paper, we focused on improving the partitioning criteria of decision trees by introducing generalized entropy and decision penalty terms, proposing new decision tree algorithms called RSE and RSEIM. The generalized entropy serves as an extension of traditional partitioning criteria, inheriting their strengths while enhancing flexibility to accommodate a wider range of datasets. The decision penalty term is an enhancement based on two information-measuring methods, considering attribute types and introducing adjustable penalty coefficients. Theoretically, RSE and RSEIM are expected to outperform the algorithms before the enhancement. These enhanced algorithms contain multiple free parameters, and we utilized a genetic algorithm to optimize these parameters in pursuit of higher classification accuracy. Experiments conducted across various datasets demonstrate a significant improvement in accuracy with RSE and RSEIM algorithms compared to traditional decision tree algorithms. Notably, the constructed trees did not exhibit increased complexity despite the improvements achieved by the enhanced algorithms.

In fact, compared with decision tree algorithms, random forests, which are ensembles of decision trees, are now more widely used in machine learning. Random forests do have stronger robustness and stability than individual trees, and they can evaluate the importance of features. In research on optimizing random forest algorithms, there are optimizations in sampling methods, optimizations targeting errors, etc., but there are relatively few studies from the perspective of optimizing the individual trees that constitute the forest. The individual decision trees optimized from the perspective of splitting criteria introduced in this paper may become a new direction for optimizing random forests. Moreover, considering the entropy-based splitting of features can provide more reference information for the final feature importance ranking.

## Figures and Tables

**Table 1:** Example for the two-term information method.

Index	$X_{1}$	$X_{2}$	*Y*
1	1	1	*A*
2	2	1	*A*
3	3	1	*A*
4	4	2	*B*
5	5	2	*B*
6	6	2	*B*

**Table 2:** Example for the improved two-term information method.

Index	$X_{1}$	$X_{2}$	*Y*
1	1	1	*A*
2	2	1.1	*A*
3	3	1.2	*A*
4	4	2	*B*
5	5	2.1	*B*
6	6	2.2	*B*

**Table 3:** Datasets for experiments.

Dataset	Instances	Attributes	Type	Classes
Scale	625	4	categorical	3
Endgame	958	9	categorical	2
Glass	214	9	numerical	6
Car	1728	6	categorical	4
Survival	306	3	numerical	2
Blood	748	4	numerical	2
Wine	178	13	numerical	3
ILPD	579	10	mixed	2

**Table 4:** Results of RSEIM algorithm.

Dataset	*r*	*s*	λ	μ	Accuracy
Scale	2.426633	1.269012	0	0.080024	0.756272
Endgame	2.660978	2.442661	0	0.013287	0.950161
Glass	0.507385	1.919376	0.602849	0	0.749374
Car	0.469276	5.238953	0	0.089872	0.972075
Survival	4.691873	5.109092	0.704681	0	0.735141
Blood	3.519761	9.278652	0.204536	0	0.723607
Wine	1.245844	9.278785	0.512542	0	0.948454
ILPD	5.900652	7.233041	0.044283	0.066182	0.728268

**Table 5:** Accuracy of different algorithms.

Dataset	GN	SE	TE	RSE	RSEIM
Scale	0.71090	0.72426	0.72037	0.75461	0.75627
Endgame	0.92527	0.92365	0.93527	0.9357	0.95016
Glass	0.64331	0.74456	0.72917	0.69987	0.74937
Car	0.95286	0.96523	0.96511	0.96845	0.97208
Survival	0.72211	0.69641	0.72211	0.73514	0.73514
Blood	0.68649	0.66380	0.70786	0.72261	0.72361
Wine	0.89237	0.89831	0.90414	0.93386	0.94845
ILPD	0.65442	0.65122	0.68579	0.72527	0.72827

**Table 6:** Results of RSEIM algorithm.

Dataset	*r*	*s*	λ	μ
TE	2.2	-	-	-
RSE	2.2	2.4	-	-
RSEIM	2.2	2.4	0.2	0

**Table 7:** VIM under different noise intensities.

Noise SD	GN		SE		TE		RSE		RSEIM	
	X	Y	X	Y	X	Y	X	Y	X	Y
0	0.5	0.5	0.5	0.5	0.5	0.5	0.91	0.09	0.13	0.87
0.05	0.46	0.54	0.45	0.55	0.47	0.53	0.92	0.08	0.18	0.82
0.1	0.58	0.42	0.4	0.6	0.46	0.54	0.88	0.12	0.25	0.75
0.15	0.61	0.39	0.58	0.42	0.44	0.56	0.89	0.11	0.29	0.71
0.2	0.45	0.55	0.52	0.48	0.45	0.55	0.85	0.15	0.31	0.69
0.25	0.54	0.46	0.53	0.47	0.52	0.48	0.78	0.22	0.38	0.62
0.3	0.55	0.45	0.48	0.52	0.52	0.48	0.75	0.25	0.45	0.55

## Data Availability

The original contributions presented in this study are included in the article. Further inquiries can be directed to the corresponding author.
